# A novel 3R-compliant *ex vivo* approach to assess ectoparasiticide repellency against *Phlebotomus perniciosus* in dogs

**DOI:** 10.1051/parasite/2026029

**Published:** 2026-06-03

**Authors:** Ambre Sibari, Guillem Weis-Servat, Emmanuel Liénard, Ali Salem, Thomas Blondel, Marie Varloud, Emilie Bouhsira

**Affiliations:** 1 CEVA Santé Animale 8 rue Logrono 33500 Libourne France; 2 Université de Toulouse, ENVT, INRAE, InTheres 23 Chemin des Capelles 31076 Toulouse France

**Keywords:** Dog, Canine Leishmaniosis, Sand flies, Membrane feeding, Effectiveness, Clinical study

## Abstract

Ectoparasiticides are essential for preventing insect bites and transmission of arthropod-borne pathogens such as *Leishmania* spp. Traditionally, their anti-feeding efficacy is evaluated *in vivo* using sedated dogs exposed to sand flies. To comply with 3R principles (replacement, reduction and refinement) and to avoid animal exposure, this study aimed to develop an *ex vivo* feeding model to assess the efficacy of a dinotefuran, permethrin, and pyriproxyfen (DPP) [Vectra^®^3D] combination against *Phlebotomus perniciosus* as an alternative to *in vivo* testing. Twelve dogs were assigned to either a DPP-treated group (*n* = 6) or an untreated control group (*n* = 6). On days -7, 1, 7, 14, 21, and 28, dogs were sedated and exposed for one hour to 50 (±5) female sand flies. In parallel, hair collected from each dog was used in an *ex vivo* artificial feeding system. After exposure, sand flies were assessed for feeding and survival rates. Anti-feeding efficacy was the primary criterion, while insecticidal efficacy was evaluated as a secondary outcome. In both models, control groups showed feeding and survival rates above 31% and 90%, confirming a reliable challenge. The treated group showed significantly lower feeding and survival rates than the control group (*p* < 0.05). In both models, DPP demonstrated a strong anti-feeding effect in dogs for one month, with efficacy above 80%. This first direct comparison of *in vivo* and *ex vivo* models confirms DPP’s fast and lasting protection against *Ph. perniciosus* and positions the *ex vivo* model as a promising 3R-aligned alternative for studying vector-borne disease prevention.

## Abstract (fr)

Les antiparasitaires externes sont essentiels pour prévenir les piqûres d'insectes et la transmission d'agents pathogènes transmis par les arthropodes, tels que *Leishmania* spp. Traditionnellement, leur efficacité anti-gorgement est évaluée *in vivo* sur des chiens anesthésiés exposés à des phlébotomes. Afin de respecter les principes des 3R (remplacement, réduction et raffinement) et d'éviter l'exposition des animaux, cette étude visait à développer un modèle de gorgement *ex vivo* pour évaluer l'efficacité d'une combinaison de dinotéfurane, de perméthrine et de pyriproxyfène (DPP) [Vectra^®^3D] contre *Phlebotomus perniciosus*, en alternative aux tests *in vivo*. Douze chiens ont été répartis en deux groupes : un groupe traité au DPP (*n* = 6) et un groupe témoin non traité (*n* = 6). Les jours -7, 1, 7, 14, 21 et 28, les chiens ont été sédatés et exposés pendant une heure à 50 (±5) phlébotomes femelles. Parallèlement, des poils prélevés sur chaque chien ont été utilisés dans un système d'alimentation artificielle *ex vivo*. Après l'exposition, les taux de gorgement et de survie des phlébotomes ont été évalués. L’efficacité répulsive (ou anti-gorgement) était le critère principal, tandis que l'efficacité insecticide était évaluée comme critère secondaire. Dans les deux modèles, les groupes témoins ont présenté des taux de gorgement et de survie supérieurs à 31 % et 90 %, respectivement, confirmant ainsi la fiabilité du modèle. Le groupe traité a présenté des taux de gorgement et de survie significativement inférieurs à ceux du groupe témoin (*p* < 0,05). Dans les deux modèles, le DPP a démontré un puissant effet anti-gorgement chez les chiens pendant un mois, avec une efficacité supérieure à 80 %. Cette première comparaison directe des modèles *in vivo* et *ex vivo* confirme la protection rapide et durable du DPP contre *Ph. perniciosus* et positionne le modèle *ex vivo* comme une alternative prometteuse, conforme aux principes des 3R, pour l'étude de la prévention des maladies vectorielles.

## Introduction

Leishmaniosis is a deadly vector-borne disease spreading on different continents, including Europe. Both humans and animals can be affected by the disease that can be fatal in immunocompromised patients and in untreated susceptible dogs. The pathogen responsible for leishmaniosis is a protozoan called *Leishmania* that is mainly transmitted through the bites of female phlebotomine sand flies (Diptera: Psychodidae) [2, 26]. To complete their life cycle, phlebotomine females require blood meals. They are hematophagous for the development and maturation of their eggs, feeding multiple times during the vectorial season, which impacts pathogen maturation and transmission [30]. One of the first lines of canine leishmaniosis (CanL) prevention is protection against vector bites. Repellent formulations with anti-feeding efficacy have been developed specifically for dog protection. To assess the effectiveness of these products, experimental dogs are traditionally exposed to numerous and repeated vector bites in controlled conditions. Alternative artificial models have been developed to reproduce the natural conditions of blood feeding for parasites [7] such as ticks [10, 31], fleas [5], mosquitoes [12, 19] and sand flies [13, 16, 21, 35]. The first membrane feeding model for sand flies was described in 1927 with rabbit skin [1]. Over the years, different models have been developed to characterize sand fly feeding behavior and to stimulate factors such as blood source [14] or temperature [9]. Artificial feeders were also historically tested to study infection by and transmission of *Leishmania* spp. [13, 22, 35, 37]. However, the only direct comparison between *ex vivo* and *in vivo* models was performed on hamsters [27] and no alternative to the dog *in vivo* infestation model has yet been developed to assess the efficacy of an ectoparasiticide against *Ph. perniciosus*, more in alignment with the 3Rs principle (reduce, refine, replace). The aim of this study was therefore to compare an *in vivo* and an *ex vivo* model of sand fly infestation in dogs through the efficacy assessment of a repellent product.

## Materials and methods

### Ethics statement

The study was conducted after approval by the ethics committee at the National Veterinary School of Toulouse, France (APAFIS#21563-2019071916306501 v6). The study complied with Good Clinical Practices (VICH GL9, June 2000). All technicians were blinded to the groups, except for the personnel involved in treatment and dog follow-up post treatment.

### Sand fly *Phlebotomus perniciosus* colony

The sand fly *Phlebotomus perniciosus* strain used in this study originated from Lisbon, Portugal, and had been maintained under laboratory conditions at École Nationale Vétérinaire de Toulouse (ENVT), France, for 20 years without being exposed to any insecticide. The sand fly females are fed on rabbit blood, and the larvae are maintained on a specific medium. The cycle from egg to adult lasts for 5–7 weeks, at optimal conditions of ambient temperature (25–30 °C) and relative humidity (70–80%). Sand fly exposures described below were performed using unfed females aged between 8 to 10 days, according to our internal procedures.

### Experimental dogs

Fourteen healthy Beagle dogs, males (5) and females (9), originating from the kennel of the Parasitology Department of the ENVT were enrolled in the study. They had not been treated with any ectoparasiticide and/or repellent within 3 months before the start of the animal phase. Prior to allocation, all dogs underwent a physical examination performed by the study veterinarian to ensure their health status. Dogs were housed by pair of 2 in indoor enclosures in compliance with European animal welfare regulations, under controlled environmental conditions with a 12-hour light/12-hour dark cycle. Each animal was identified by a subcutaneously implanted microchip. They received a commercially available dry diet in appropriate daily rations and had *ad libitum* access to water. All animals were handled and maintained with due consideration for their welfare and were acclimated to the facility for two weeks prior to the initiation of treatment. Throughout the study period, the dogs were monitored daily for general health status and remained in good health during the entire animal phase.

### Dog study design

**Figure 1:**
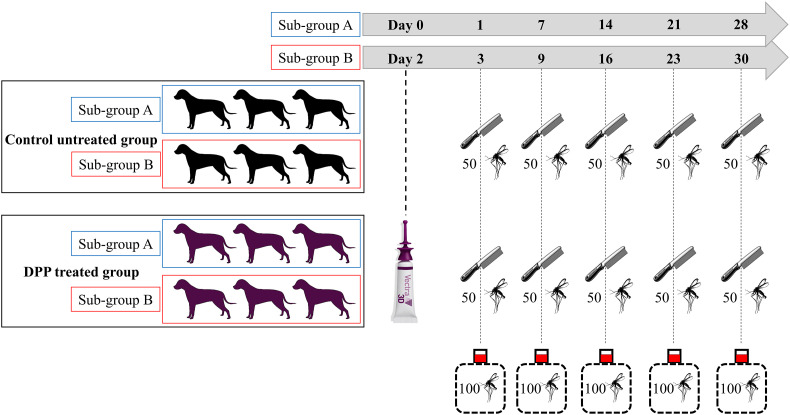
Study design diagram. 12 dogs were allocated to either an untreated control group or a treated group receiving Vectra 3D^®^. Each group was further divided into two sub-groups (A and B) to allow staggered experimental procedures. Treatment was performed on Days 0 and 2 for sub-groups A and B, respectively. Dogs were combed for hair, which was then used for assessments performed on Days 1, 7, 14, 21, and 28 post-treatment for sub-group A and on Days 3, 9, 16, 23 and 30 post-treatment for sub-group B.

The study was a randomized, controlled, unicenter trial with a parallel design. The study followed a randomized block design based on the number of engorged (dead + alive) *Ph. perniciosus* sand flies collected from each dog after the pre-treatment infestation. The pre-infestation phase was conducted with 14 dogs, from which the two dogs with the lowest number of engorged females were removed, leaving 12 dogs for the trial. These twelve dogs (12.9 ± 1.3 kg body weight) were then allocated into 6 replicates of 2 animals each. The two dogs with the highest number of engorged *Ph. perniciosus* (dead + alive) females formed replicate 1, the next 2 formed replicate 2, and so on. Within each replicate, dogs were randomly assigned to one of two groups: a Dinotefuran-Permethrin-Pyriproxyfen [DPP, VECTRA^®^ 3D] treated group (*n* = 6) or an untreated control group (*n* = 6) (Figure 1). To facilitate the workload for both *in vivo* and *ex vivo* infestations over time, each group was further divided into two sub-groups A and B: the first 3 dogs were allocated to sub-group A, and the remaining 3 to sub-group B.

### *In vivo* model

Infestations of dogs were performed as previously described [17]. The first infestation was conducted prior to treatment at day-7 and was used for allocation purposes. Five post-treatment infestations were conducted at 1, 7, 14, 21, and 28 days post-treatment. The day before each infestation, 50 (±5) *Ph. perniciosus* females and approximately 5 males were placed in each sand fly-proof net, with cotton soaked in sugar-water at their disposal. Sand flies were fasted for 2 h before exposure to the dogs. Prior to infestation, animals were weighed and sedated by intramuscular injection of a mixture of dexmedetomidine (at the recommended dose of 25 μg/kg) (Dexdomitor^®^, Elanco Santé Animale, Lilly, Suresnes, France) and ketamine (at the recommended dose of 5 mg/kg) (Clorketam^®^, Laboratoire Vetoquinol S.A., Lure, France) and diazepam (at the recommended dose of 0.45 mg/kg) (Valium^®^) to facilitate muscular relaxation, following the dose recommended by the manufacturers, and then placed in each individual infestation net. After 60 (±5) minutes of exposure, dogs were carefully taken out of the nets and examined for any dead sand flies, then placed back in their individual boxes and supervised until they regained full consciousness. At the end of the exposure period, all live sand flies were aspirated from the nets using a vacuum pump and recorded as live-engorged or live-unengorged. All dead sand flies found on the dog’s body or in the net were collected, counted, and recorded as dead-unengorged or dead-engorged. Engorgement status was determined by observing abdominal distension and the presence of blood with the naked eye. To facilitate counting, live sand flies recovered from individual animals after exposure were placed in separate net cages (one net per animal). Each cage was labelled with the animal number. Once the counting of alive sand flies was completed, all remaining sand flies were discarded.

### *Ex vivo* model

#### Hair collection

The hair specimens were collected from each individual dog by combing the whole body until approximately 1 g of hair was obtained per dog. The hair collected from each dog was then placed into aluminium foil and stored in an individual plastic bag identified with the dog’s number, the date, and, when relevant, the treatment group number. Hair combing was performed once on Day-7 and then after treatment and before anesthesia, on Days 1, 7, 14, 21, and 28 for dogs from Groups 1A and 2A, and on Days 3, 9, 16, 23, and 30 for dogs from Groups 1B and 2B.

#### Infestation

The day before each infestation, 100 (±5) *Ph. perniciosus* females and approximately 5 males, between 8 and 10 days old, were placed in each small sand fly-proof net, with cotton soaked in sugar-water at their disposal. Each net was identified with the corresponding individual number of each artificial feeder. Sand flies were fasted for approximately two hours before exposure to the feeders.

**Figure 2:**
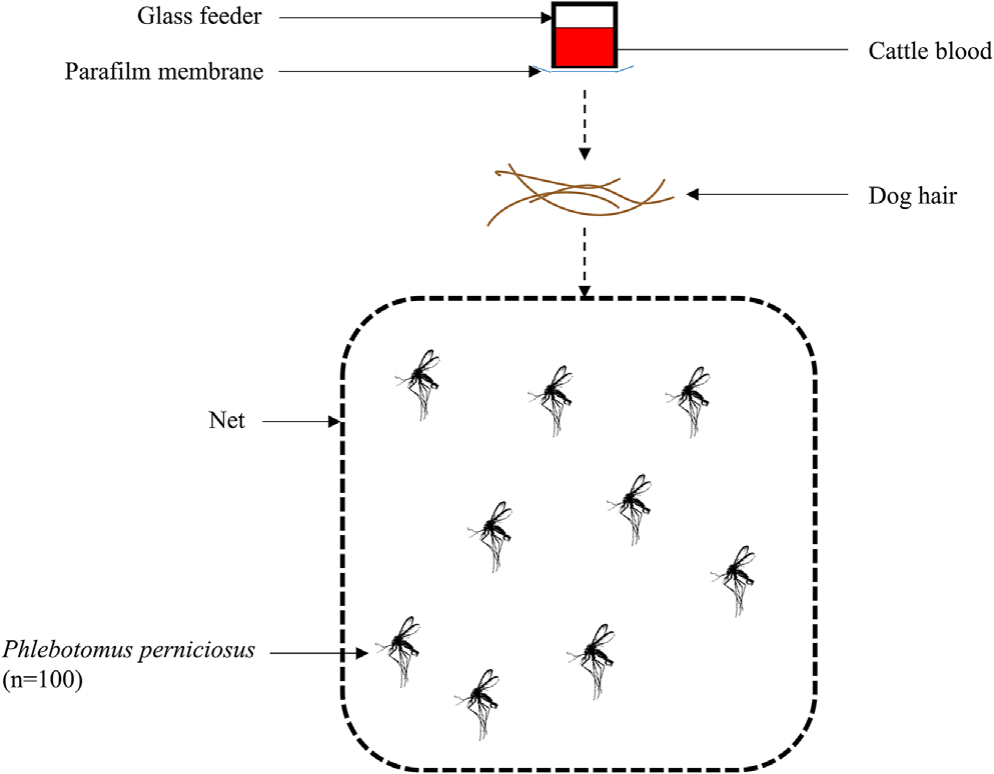
Diagram representation of the *ex vivo* feeding system. The system is composed of a glass feeder containing cattle blood and covered at its base with a synthetic membrane. Dog hair samples were placed between the feeder and a net enclosing approximately 100 *P. perniciosus* sand flies, allowing assessment of feeding under *ex vivo* conditions.

The individual glass feeders were closed at the bottom with a thin Parafilm membrane (Parafilm^®^ M; Pechiney Plastic Packaging, Chicago, IL, USA) and identified with the corresponding individual number of each dog (one glass feeder represented one dog). They were filled with cattle blood derived from residual clinical samples collected at the ENVT Large Animal Hospital, treated with lithium heparin (approximately 5 mL to cover the Parafilm membrane). To stimulate the sand flies to feed on the device, a constant temperature of 38.5 °C (±0.5 °C), mimicking the host’s body temperature, was maintained by water circulating through the glass feeder. Once the blood was added and the water system started, approximately 0.09 g of hair (corresponding to 0.007 g of hair/cm^2^ following the *ex vivo* model from Tahir *et al*. [31]) collected from each dog was placed on top of each individual net, and the corresponding artificial feeder (4 cm in diameter, surface of approximately 12.6 cm^2^) was placed on top of it, allowing the sand flies to feed directly onto the blood through the hair and the Parafilm membrane + surface feeder (Figure 2). The system was left for 60 min (±5 min). At the end of the exposure period, all sand flies were counted and recorded as live/dead and engorged/unengorged.

The sand flies were exposed to the artificial devices on Day-7, and then on Days 1, 7, 14, 21, and 28 for dogs from Groups A and on Days 3, 9, 16, 23, and 30 for dogs from Groups B.

#### Product efficacy assessment

The product efficacy was assessed based on various criteria such as the feeding rate and survival rate of sand flies, calculated for each dog at each time point. The primary efficacy criterion was the anti-feeding (also called repellent) effect. The 1-hour insecticidal efficacy against the total number of sand flies and the 1-hour insecticidal efficacy against fed sand flies only were determined as secondary efficacy criteria.

Feeding rate (%):

$$100\times \frac{\text{Total number of}\;\mathrm{fed}\;\text{sand flies}}{\text{Theoretical number of sand flies released}\;}.$$

Anti-feeding efficacy (%):

$$100\times \frac{\mathrm{MC}-\mathrm{MT}}{\mathrm{MC}},$$

where MC and MT are the arithmetic mean of engorged sand flies in the control and treated groups, respectively.

Survival rate (%):

$$100\times \frac{\text{Total number of live sand flies}}{\;\text{Theoretical number of sand flies released}\;}.$$

Insecticidal efficacy (%):

$$100\times \frac{\mathrm{MC}-\mathrm{MT}}{\mathrm{MC}},$$

where MC and MT are the arithmetic mean of live sand flies in the control and treated groups, respectively.

Data were entered using Excel and statistical analysis was performed using R software R version 4.0.5 (https://www.R-project.org/.) The parameters were compared at each time point between control and treated groups, as well as between the *in vivo* and *ex vivo* models for each dog. A Wilcoxon test was applied. A *p*-value of <0.05 was considered statistically significant. Multiple comparisons were performed using the Holm adjustments to manage multiplicity testing.

## Results

The product was well-tolerated by the dogs and no adverse reactions were observed. Our study was aimed to determine whether the effect of the treatment was comparable when using the reference *in vivo* dog model vs. an alternative *ex vivo* model.

### Feeding rate and survival rate of sand flies *in vivo*

#### Feeding rate of sand flies on dogs

The average feeding rate of sand flies on dogs was 82.5 ± 6.9% in the control group. In the treated group, the feeding rate on dogs ranged between 2% (day 7) to 15% (day 28). On day 28, an outlier female dog in the treated group was identified with a feeding rate of 46%. At each time point post-treatment, the difference in the feeding rate status of *Ph. perniciosus* females was higher (*p* < 0.01) in the control than in the treated group (Table 1).

**Table 1:** Comparative analysis of feeding and survival rates of sand flies (± standard deviation) *in vivo* over time.

	Group	D-7	D1	D7	D14	D21	D28
Feeding rate of sand flies	Control	84.0 ± 7.6	78.0 ± 8.9	85.0 ± 6.8	83.0 ± 7.0	79.0 ± 4.1	87.0 ± 4.8
	Treated		4.0 ± 6.9	2 ± 2.2	6 ± 2.8	5.6 ± 0.9	15 ± 15.9
*p*-value			0.021	0.021	0.021	0.021	0.021
Survival rate of sand flies	Control	89.3 ± 8.6	90.0 ± 5.4	95.7 ± 2.7	92.0 ± 3.7	93.0 ± 3.3	93.0 ± 3.5
	Treated		17.0 ± 14.1	22.0 ± 14.0	38.0 ± 17.0	38.4 ± 18.1	55 ± 24.9
*p*-value			0.023	0.023	0.023	0.023	0.023

#### Survival rate of sand flies after exposure to dogs

The average survival rate of sand flies on dogs was 92.6 ± 4.0 % in the control group. In the treated group, the survival rate on dogs ranged between 17% (day 1) to 55% (day 28). On day 28, the outlier female dog was identified in the treated group with a survival rate of 70%. At each time point post-treatment, the survival rate of *Ph. perniciosus* females was higher (*p* < 0.01) in the control than in the treated group (Table 1).

### Feeding rate and survival rate *ex vivo*

#### Feeding rate of sand flies on membranes

The average feeding rate of sand flies on membranes was above 40.1 ± 15.2 % in the control group (Table 2). In the treated group, the feeding rate ranged between 1% (day 1) to 4% (day 21). At each time point post-treatment, the difference in the feeding rate status of *Ph. perniciosus* females was higher (*p* < 0.01) in the control than in the treated group (Table 2).

**Table 2:** Comparative analysis of feeding and survival rates of sand flies (± standard deviation) ex vivo over time.

	Group	D-5	D1	D7	D14	D21	D28
Feeding rate of sand flies	Control	49.7 ± 4.9	51.0 ± 18.6	31.0 ± 16.2	31.0 ± 12.1	40.0 ± 11.8	47.0 ± 5.8
	Treated		3.0 ± 5.0	1 ± 1.6	1.0 ± 2.0	2 ± 1.9	4 ± 4.4
*p*-value			0.019	0.018	0.019	0.01	0.019
Survival rate of sand flies	Control	85.7 ± 6.3	94.0 ± 3.8	98.3 ± 1.4	97.0 ± 2.3	98.0 ± 4.1	97.0 ± 2.2
	Treated		51.0 ± 14.4	72.5 ± 11.7	62.0 ± 8.7	70 ± 12.6	77 ± 9.6
*p*-value			0.023	0.02	0.023	0.02	0.023

#### Survival rate of sand flies after exposure to membranes

The average survival rate of sand flies on dogs was above 96.7 ± 3.2 % in the control group (Table 2). In the treated group, the survival rate ranged between 51% (day 28) to 77% (day 21). At each time point post-treatment, the survival rate of *Ph. perniciosus* females was higher (*p* < 0.01) in the control than in the treated group (Table 2).

#### Efficacy assessment

**Figure 3:**
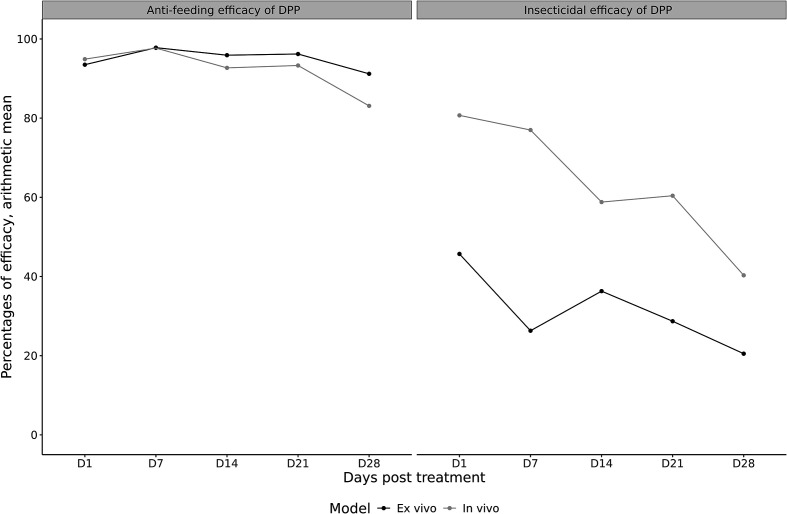
Comparison of anti-feeding and insecticidal efficacy (arithmetic mean) of Vectra 3D^®^ between *in vivo* & *ex vivo* models. Anti-feeding efficacy (left panel) and insecticidal efficacy (right panel) are presented as arithmetic means at each time point (Days 1, 7, 14, 21, and 28 post-treatment) for both *ex vivo* and *in vivo* models. Efficacy was calculated relative to the untreated control group.

**Figure 4:**
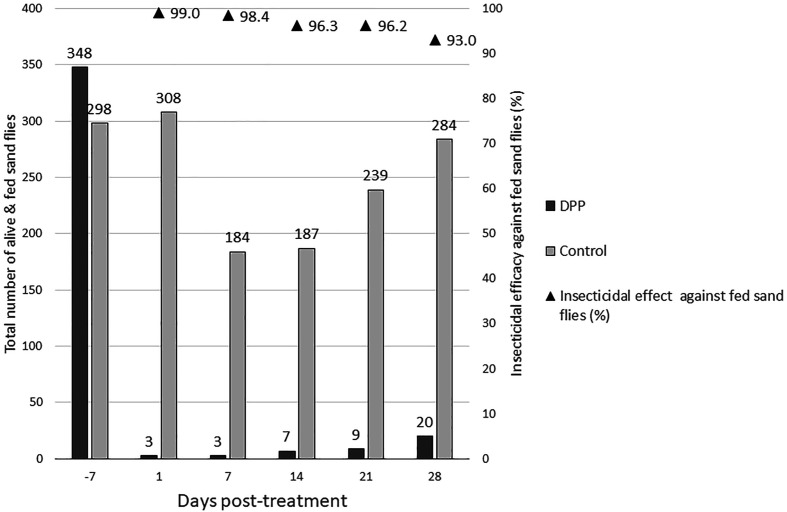
Insecticidal efficacy against fed sand flies with the *in vivo* model. The total number of alive and fed sand flies in the *in vivo* model is shown for the untreated control and treated groups at each time point (Day -7, and Days 1, 7, 14, 21, and 28 post-treatment). Anti-feeding efficacy (%) is indicated above the bars and represented by black triangles.

**Figure 5:**
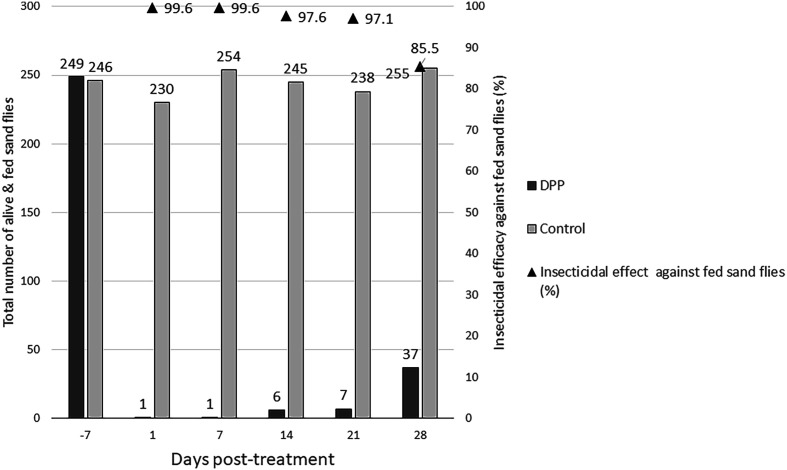
Insecticidal efficacy against fed sand flies with the *ex vivo* model. The total number of alive and fed sand flies in the *ex vivo* model is shown for the untreated control and treated groups at each time point (Day -7, and Days 1, 7, 14, 21, and 28 post-treatment). Anti-feeding efficacy (%) is indicated above the bars and represented by black triangles.

**Supplementary Figure 1:**
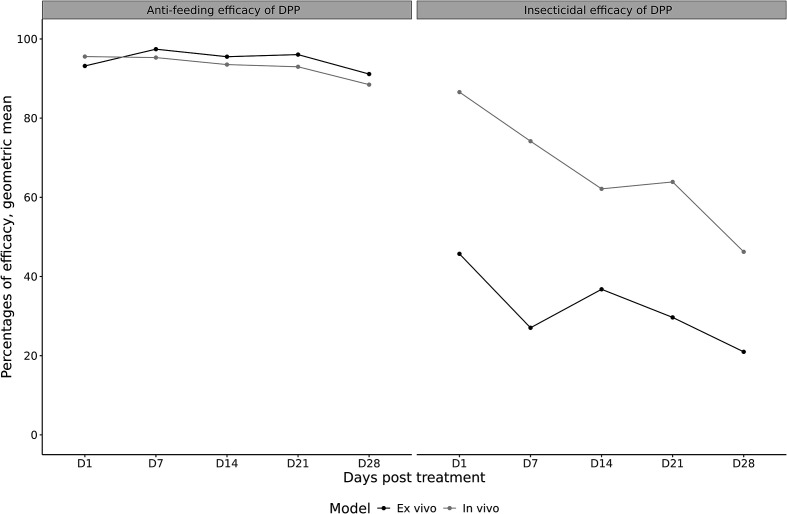
Anti-feeding efficacy (left panel) and insecticidal efficacy (right panel) are presented as geometrical means at each time point (Days 1, 7, 14, 21, and 28 post-treatment) for both the ex vivo and in vivo models. Efficacy was calculated relative to the untreated control group.

In the treated group, the application of DPP demonstrated an anti-feeding effect in dogs lasting for one month, with efficacy exceeding 83% (Table 3). Similar results were observed with the artificial feeder, achieving anti-feeding efficacy greater than 91% over time. For both models, the onset of efficacy was observed within 24 h of product administration (D1) and maximum efficacy was reached at Day 7 with an anti-feeding effect above 97%. The insecticidal effect was also evaluated in both models (Table 3). In the *in vivo* model, efficacy ranged from 80.7% on Day 1 to 40.3% on Day 28, whereas in the *ex vivo* model it ranged from 45.7% (Day 1) to 20.5% (Day 28) (Table 3). A comparison of anti-feeding and insecticidal efficacies between the two models is presented in Figure 3 and Supplementary Figure 1. In addition, the insecticidal efficacy of the product against fed sand flies was evaluated, with results presented in Figures 4 and 5.

**Table 3:** Evaluation of anti-feeding and insecticidal efficacy (%) of Vectra 3D^®^ against sand flies from D1 to D28.

Parameter	Model	D1	D7	D14	D21	D28
Anti-feeding efficacy of DPP	*In vivo*	94.9	97.7	92.7	93.	83.1
	*Ex vivo*	93.5	97.8	95.9	96.2	91.2
Insecticidal efficacy of DPP	*In vivo*	80.7	77.0	58.8	60.	40.3
	*Ex vivo*	45.	26.3	36.3	28.	20.5

## Discussion

The objective of this study was to develop an alternative model to dog infestations by sand flies used for the efficacy assessment of parasiticide repellent products. To the best of the authors’ knowledge, this work documents for the first time a direct comparison between *in vivo* and *ex vivo* models of sand fly infestation in dogs for the assessment of ectoparasiticidal product efficacy.

### Methodological considerations

The methodology is a key success factor in the development of this alternative *ex vivo* model. In this study, certain methodological aspects affected model performance and some could be improved. We will discuss the impact of methodological choices on both feeding and survival rates, such as the number of sand flies, the type of membrane, the exposure time, and the feeding assessment of the sand flies.

### Feeding rate

In the present study, we observed that the mean feeding rate in the untreated control group using the *in vivo* model (82.5% ±6.9) was 49% higher than with the *ex vivo* model (40.1% ±15.2), suggesting that the current methodology could be further optimized. Importantly, the feeding rate is generally considered satisfactory for inclusion of dogs prior to treatments when it is above 50% [17]. This improvement should be achievable as a previous comparison between *in vivo* (hamster, >60%) and *ex vivo* (membrane, >70%) did not detect significative differences in feeding rates of sand flies between the two models [27].

### Number of sand flies

The number of sand flies per challenge in this study (50 *in vivo*, 100 *ex vivo*) may have influenced the feeding rate. Of note, a study has demonstrated a positive correlation between the number of phlebotomine sand flies and the feeding rate [29]. Evidence suggests that cooperative feeding improves feeding efficiency, as single-feeding flies take 70.5% longer to obtain a blood meal compared to those feeding in groups [32]. This cooperative behavior significantly influences sand fly behavior during blood meals and affects *Leishmania* transmission and development by enhancing the effectiveness of saliva components [32]. However, this parameter might be dependent on other factors. Similarly, in a previous study [17], the use of twice the number of female sand flies released with dogs (100 *vs*. 50) did not lead to a higher absolute value feeding rate (74%) than in our study with the *in vivo* model (82%). The number of sand flies should also be considered with the surface or volume available in the model. While this would not be limiting with the *in vivo* model using the full body of dogs, it might represent a limiting factor with the *ex vivo* model. The optimum ratio of sand flies to surface area or volume remains a critical parameter for understanding sand fly feeding behavior. In previous studies, different feeding surface areas of 3.14 cm^2^ [37] and 7.1 cm^2^ [29] were used, but the number of sand flies was not well documented. In the present study, the surface area available for sand flies to feed on in the *ex vivo* model was approximately 12.6 cm^2^.

### Type of membrane and blood source

A parafilm membrane was chosen in this study as the non-animal membrane and its application for artificial blood feeders is well-documented in the literature [8, 11, 15]; it has been used extensively in other *in vitro* systems developed by the ENVT collaborators with other insect models [6, 18]. In studies comparing different membrane compositions, animal membranes such as chicken skin membranes were the most successful for sand fly feeding in species such as *Lutzomyia longipalpis* [37], *Ph. papatasi* [9, 27], and *Ph. perniciosus* [15]. Hošková *et al.* compared different membrane types in a recent study, and the results showed that only 5% of *Ph. perniciosus* females fed through a synthetic membrane, compared to 55.7% with a chicken skin membrane. However, feeding rates increased to 25.5% with the synthetic membrane when coagulated blood plasma was applied to its outer surface [15].

In a previous study [27], the authors compared chicken-skin membrane with an *in vivo* model (live hamster) and the results showed no difference between both models. However, the use of animal skins poses ethical question as they are euthanized for the methodology [29]. In the same study, the relationship between membrane thickness and feeding rate was investigated, but the results demonstrated no correlation [29].

Cattle blood was provided as a blood source for the *ex vivo* model. Previous research has shown that the origin of the blood, whether from animals, humans, or a mixture does not influence the feeding rate of other species such as *Ph. papatasi* [9, 14]. Furthermore, the ENVT sand fly colony is occasionally fed with cattle blood, in addition to rabbit blood, for colony maintenance. Additionally, dog hair was applied to the membrane system to act as a mechanical and chemical stimuli, imitating the presence of the host animal to trigger their feeding behavior.

### Exposure time

Exposure time is also a parameter to consider regarding the difference in feeding rate between the two models. In this study, the sand flies were left to feed for approximately 1 h on the *ex vivo* model, but this length of time was questioned in a previous study comparing two models with live hamster and *Ph. papatasi* species [27]. In the study, the researchers extended the initial exposure time of sand flies with the *ex vivo* model by up to 3 h of additional time to optimize engorgement and observed no significant difference between the two methods. During the present study, the feeding behavior of female sand flies was also monitored. It was observed that females typically initiated feeding within the first 30 min of exposure to both the live animal hosts and the membrane feeding system (authors’ personal observations). Consequently, extending the duration of exposure to the feeding system would likely not have resulted in a significant increase in the overall feeding rate within the control group.

### Feeding assessment

In this study, feeding was determined by visual assessment of the abdominal distension of sand flies and its change of color. This method is easy, rapid, and cost-effective to perform. However, the visual assessment is subjective and may not detect a small bloodmeal. The mean blood volume taken by *Ph. perniciosus* females from live rabbits has been estimated to be 0.51 μL in a previous study [36], but this is generally detected without microscopic examination, with the naked eye. It remains unknown whether partial blood meals occur and whether they can be reliably detected through visual assessment alone. Molecular techniques, such as real-time PCR and ELISA, are available and allow for accurate identification of phlebotomine sand fly blood meals [20, 28]. The detection limit of host DNA using the real-time PCR method for a 2 μL sand fly blood meal varied depending on the host species, with a threshold of 1 pg for canine blood [28]. Future studies should consider combining visual inspection with molecular approaches to more precisely evaluate feeding rates.

### Survival rate

The viability of sand flies is a critical element of their feeding activity, as a higher survival rate directly correlates with an increased feeding rate, allowing for more frequent and successful blood meals. In the present study, the survival rates in the control group with the *in vivo* model remained above 90%, which is consistent with other product trials [4, 24, 25], while it was >94% with the *ex vivo* model.

### Product efficacy

The main goal of repellent parasiticide products is to protect dogs from the bites of sand flies. This is demonstrated through the anti-feeding efficacy. To a lesser extent, these products usually also kill sand flies through their insecticidal efficacy. For both parameters, it is important to document the onset of action, which is the time interval between treatment and first documentation of efficacy, and the duration of this efficacy over time, which will indicate the treatment intervals.

### Anti-feeding efficacy

Regardless of the model, the overall anti-feeding effect of DPP for one month measured in this study against *Ph. perniciosus* sand flies was above 83% and was consistent with previous trials using *in vivo* models showing efficacy >83% [17] and >92% [33, 34]. The onset of anti-feeding efficacy was documented 24 h after administration at 94.9% in dogs and 93.5% *ex vivo*. In previous studies, within 24 h of administration, we observed with DPP similar anti-feeding efficacy above 80.6% [3] and 96.9% [17].

The duration of the repellent effect of DPP over time was 4 weeks with a maximum reached at Day 7 with both *in vivo* an *ex vivo* models: 97.7% and 97.8%, respectively. The study confirmed that DPP protects dogs from sand fly bites for at least 4 weeks.

### Insecticidal efficacy

Because of its strong repellent effect, the product prevents most sand flies from landing and from remaining on dogs. Therefore, their exposure to the treated animal is not long enough to trigger a repeatable and strong insecticidal effect. In fact, it was demonstrated in a previous study that contact is required between treated dogs and vectors to induce insecticidal effect [23]. The insecticidal efficacy remained above >40.3% with the *in vivo* model, while this value was above >20.5% with the *ex vivo* model. The difference could be explained by a possible higher attraction from the whole dog body compared to the membrane with hair.

In this study, the onset of insecticidal action of DPP was assessed 24 h after administration at 80.7% in dogs and 45.7% *ex vivo*. In previous studies, insecticidal efficacy greater than 97.6% was observed within 24 h of treatment with DPP in dogs [17], indicating that direct contact between treated animals and the vector is necessary for the effect to occur [23]. The insecticidal effect of DPP differed significantly between the two models, with values obtained from the *in vivo* model being at least 1.6 times higher than those observed in the *ex vivo* model.

This result showed that improvements in the methodology are achievable to obtain equivalence between the two models. The insecticidal efficacy against fed sand flies was also assessed. A fed sand fly can be infected and then transmit pathogens to another host. In the present study, the results showed that the insecticidal efficacy against fed sand flies remained above 85.5% and 93% with the *in vivo* and *ex vivo* models, respectively, over 4 weeks. These results demonstrate that the DPP product could play a significant role in global prevention against the transmission of canine leishmaniosis by efficiently controlling vector bites.

## Conclusion

This study highlights a promising *ex vivo* alternative to the traditional *in vivo* dog infestation model for evaluating the efficacy of ectoparasiticide repellents against phlebotomine sand flies. The development of this artificial model aligns with the 3Rs principles, aiming to reduce and refine the use of animals in research. While further optimization of the methodology is needed, the model has already yielded consistent efficacy results when compared to the *in vivo* reference method and to previously reported product performances [17, 33]. Additionally, regardless of the model used, the anti-feeding efficacy of DPP against sand flies reached 93.5% within 24 h post-administration and remained above 83.1% for up to one month. This *ex vivo* model should be considered by regulatory authorities as a viable tool for future efficacy assessments.

## Supplementary material

The supplementary material of this article is available at https://www.parasite-journal.org/10.1051/parasite/2026029/olm.
